# Work-Up and Treatment Strategies for Individuals with *PIK3CA*-Related Disorders: A Consensus of Experts from the Scientific Committee of the Italian Macrodactyly and PROS Association

**DOI:** 10.3390/genes14122134

**Published:** 2023-11-27

**Authors:** Andrea Gazzin, Chiara Leoni, Germana Viscogliosi, Federica Borgini, Lucrezia Perri, Matteo Iacoviello, Marilidia Piglionica, Maurizio De Pellegrin, Giovanni Battista Ferrero, Andrea Bartuli, Giuseppe Zampino, Paola Sabrina Buonuomo, Nicoletta Resta, Alessandro Mussa

**Affiliations:** 1 Clinical Pediatric Genetics Unit, Department of Public Health and Pediatrics, University of Torino, Regina Margherita Children’s Hospital, 10126 Torino, Italy; andrea.gazzin@unito.it (A.G.); alessandro.mussa@unito.it (A.M.); 2 Postgraduate School of Pediatrics, University of Torino, 10126 Torino, Italy; 3 Center for Rare Diseases and Birth Defects, Department of Woman and Child Health and Public Health, Fondazione Policlinico Universitario A. Gemelli, IRCCS, 00168 Rome, Italy; germana.viscogliosi01@icatt.it (G.V.); lucrezia.perri@guest.policlinicogemelli.it (L.P.); giuseppe.zampino@policlinicogemelli.it (G.Z.); 4 Italian Macrodactyly and PROS Association, 27010 San Genesio ed Uniti (PV), Italy; areascientifica@associazione-nazionale-macrodattilia.org; 5 Medical Genetics Unit, Department of Precision and Regenerative Medicine and Ionian Area (DiMePRe-J), University of Bari “Aldo Moro”, 70124 Bari, Italy; m.iacoviello3@gmail.com (M.I.); marilidia.piglionica@uniba.it (M.P.); nicoletta.resta@uniba.it (N.R.); 6 Pediatric Orthopedic Unit, Piccole Figlie Hospital, 43125 Parma, Italy; 7 Department of Orthopedics, ASST Ospedale Papa Giovanni XXIII, 24127 Bergamo, Italy; 8 Department of Clinical and Biological Sciences, University of Torino, 10126 Torino, Italy; giovannibattista.ferrero@unito.it; 9 Rare Disease and Medical Genetics Unit, Bambino Gesù Children’s Hospital, IRCCS, 00168 Rome, Italy; andrea.bartuli@opbg.net (A.B.); psabrina.buonuomo@opbg.net (P.S.B.); 10 Faculty of Medicine and Surgery, Università Cattolica del Sacro Cuore, 00168 Rome, Italy

**Keywords:** *PIK3CA*, PI3K/AKT/mTOR, overgrowth, *PIK3CA*-related overgrowth spectrum, personalized medicine, repurposed drugs, target therapy

## Abstract

*PIK3CA*-related disorders encompass many rare and ultra-rare conditions caused by somatic genetic variants that hyperactivate the PI3K-AKT-mTOR signaling pathway, which is essential for cell cycle control. *PIK3CA*-related disorders include *PIK3CA*-related overgrowth spectrum (PROS), *PIK3CA*-related vascular malformations and *PIK3CA*-related non-vascular lesions. Phenotypes are extremely heterogeneous and overlapping. Therefore, diagnosis and management frequently involve various health specialists. Given the rarity of these disorders and the limited number of centers offering optimal care, the Scientific Committee of the Italian Macrodactyly and PROS Association has proposed a revision of the most recent recommendations for the diagnosis, molecular testing, clinical management, follow-up, and treatment strategies. These recommendations give insight on molecular diagnosis, eligible samples, preferable sequencing, and validation methods and management of negative results. The purpose of this paper is to promote collaboration between health care centers and clinicians with a joint shared approach. Finally, we suggest the direction of present and future research studies, including new systemic target therapies, which are currently under evaluation in several clinical trials, such as specific inhibitors that can be employed to downregulate the signaling pathway.

## 1. Introduction

*PIK3CA*-related disorders encompass several rare and ultra-rare genetic conditions caused by somatic activating pathogenic variants in the *PIK3CA* gene. The epidemiology of such disorders has been poorly studied and is likely largely underestimated worldwide: a recent study estimated the prevalence of such conditions at 1 case in approximately 22,000 live births [1].

*PIK3CA*-related disorders result from the overactivation of the intracellular PI3K-AKT-mTOR pathway consequent to gain-of-function variants in *PIK3CA*, a key player that is vital for the regulation of the cellular cycle and other major cellular functions [2]. Typically, *PIK3CA*-related disorders are caused by postzygotic variants resulting in a selective growth advantage and higher replication rate of the involved cell line. The clinical consequences of a variant depend on several factors: the gestational age when the variant occurs, the specific anatomical site affected and the type of tissue experiencing cellular overgrowth including intrinsic factors such as cell lineage susceptibility and extrinsic factors (environmental modifiers). The severity of the disease tends to be greater when the mutational event happens earlier in gestation, leading to a higher level of mosaicism [3]. The rate of overgrowth is mainly influenced by the type of variant, as different variants can have diverse activating effects. The extent and type of tissues or organs involved, along with the activating capacity (referred to as “strength” or “oncogenicity”) of the variant, ultimately determine the severity of the clinical manifestations [4]. In this context, *PIK3CA*-related disorder phenotypes are extremely heterogeneous, and it is not always easy to associate clinical findings with a specific condition. Moreover, since most of these conditions are rare, multiple medical assessments by various specialists are often required to make a proper diagnosis. A late diagnosis may be tremendously troublesome for the patient and their family/caregivers, as it delays the establishment of an appropriate care plan. Since the clinical follow-up and management strategies may significantly vary according to different countries, health care systems and specialists, the creation of common guidelines is essential to warrant the same diagnostic and therapeutic strategies as standard of care at a national level. 

With the aim of promoting collaboration between health care centers and clinicians in a shared health care management setting and to reach a national uniform approach with a joint shared approach, a careful revision of the most recent literature on *PIK3CA*-related disorders was conducted and is summarized in this paper in the form of practice recommendations. The nominal group technique was used to structure the participants’ interaction and group discussion in this review, with the aims of reaching a consensus, problem-solving, determining priorities, and generating agreement upon suggestions and recommendations provided [5].

## 2. Clinical Diagnosis

Given the complexity and heterogeneity of the phenotypes, in recent years, many authors have focused on making effort to provide a clinical classification to correctly identify the conditions belonging to PROS [2,3,6,7]. In 2021, Canaud and collaborators suggested a reclassification of the conditions caused by pathogenetic variants in *PIK3CA* [2], superseding the previous classification published in 2013 [6]. The authors defined three categories of *PIK3CA*-related conditions: (A) *PIK3CA*-realted overgrowth spectrum disorders (PROS), characterized by overgrowth of the affected tissues; (B) exclusive vascular proliferation, named *PIK3CA*-related vascular malformations; (C) exclusive non-vascular proliferation, named *PIK3CA*-related non-vascular lesions [2]. Nevertheless, given the many possible combinations of tissues and body districts affected by overgrowth, not every patient’s phenotype fits neatly into this classification. In the latter case, they may be broadly defined as “patients affected by PROS” or “patients affected by *PIK3CA*-related disorders” and have their phenotype and functional status described in detail, to plan a correct individualized follow-up strategy over time. 

Overgrowth, usually asymmetric, is the most evident clinical feature, and it may involve various parts of the body (mainly the brain, limbs, trunk, and face) and more than one type of tissue (nervous, vascular, lymphatic, skeletal, or adipose). It is usually present at birth, or it develops early in infancy.

Multiple lipomas may be present, most commonly involving the trunk, and often infiltrating the surrounding structures. They are a common cause of pain. Their more severe consequences occur when they compress the spinal cord and nerve roots or when they invade visceral organs or muscle. Typical findings in patients with variants in *PIK3CA* include limb anomalies such as leg-length discrepancy [8], sandal gap, macro-/poly-/syn-dactyly, finger and toe deformities, wide toes, and stiff fingers. Sternum and vertebrae abnormalities, joint hypermobility and laxity, scoliosis, and spina bifida are also described. Cutaneous abnormalities are another evident feature at clinical examination. In detail, epidermal nevi are often—but not always—present, as are café-au-lait spots, seborrheic keratoses, hypo-/hyperpigmented areas, benign lichenoid keratoses, acrochordons, pigmented nevi, etc. [9] Vascular anomalies are also involved in the *PIK3CA* spectrum, with capillary, lymphatic, venous, arteriovenous, and combined malformations. They can be located superficially (e.g., on a limb in association with overgrowth) or in deeper tissues. Capillary malformations may appear as well as demarcated dark red/purple patches of irregular shape, defined as “capillary malformation with geographic borders”, or as reticulated patches with poorly-defined edges, pink to light red in color, named “reticulated capillary malformations”. The former are typical of congenital, lipomatous overgrowth, vascular malformations, epidermal nevi and scoliosis/skeletal/spinal anomalies (CLOVES) syndrome and of Klippel–Trenaunay Syndrome (KTS), while the latter are characteristic of megalencephaly–capillary malformation (MCAP) and diffuse capillary malformation with overgrowth (DCMO). However, overlap of both morphologies is possible. Other vascular malformations, typical of certain phenotypes, are the persistence of marginal embryonic veins in Klippel–Trenaunay syndrome or spinal/paraspinal arteriovenous malformations in CLOVES phenotypes. Finally, the localization and type of certain vascular malformations are specific to a particular medical condition or entity, such as the persistent midfacial capillary malformation in MCAP or the capillary malformation with geographic borders of the lower lip in the capillary malformation of the lower lip, lymphatic malformation of the face and neck, asymmetry of the face and limbs, and partial or generalized overgrowth (CLAPO) syndrome. A more detailed description of vascular anomalies can be found in the SISAV (Società Italiana per lo Studio delle Anomalie Vascolari) guidelines [10] and the ISSVA (International Society for the Study of Vascular Anomalies) classification [11]. Vascular malformations may be associated with bleeding diathesis, lymphedema, cellulitis and other infections, pain, increased risk of superficial thrombophlebitis, deep vein thrombosis, and pulmonary embolism. The risk is higher after surgery, sclerotherapy, or other chronic causes of venous congestion such as reduced mobility and vascular endothelial disorders due to the pathogenic variant.

Overgrowth and other anomalies may also involve the central nervous system (CNS) with macrocephaly, megalencephaly, hemimegalencephaly, ventriculomegaly, hydrocephalus, thick corpus callosum, herniation of the cerebellar tonsils (Chiari malformation), syringomyelia, cortical dysplasia, polymicrogyria, and hypophyseal hypoplasia or pituitary stalk interruption (with or without hormonal deficiencies) being most commonly reported. When CNS involvement occurs, neurological functional impairment of variable degree is possible, such as delayed psychomotor development, intellectual disability/developmental delay (ID/DD), motor delay, behavioral and mood disorders, epilepsy (sometimes drug-resistant), respiratory symptoms, hearing loss, feeding difficulties, headache, sensory disturbances, hypotonia, muscle weakness, posture and gait disorders, vertigo, and neck pain. Patients may also present with other manifestations such as renal abnormalities (hydronephrosis, hydroureter, renal hypoplasia/aplasia, cysts), hypotonia, and ocular issues.

Some individuals may also present segmental undergrowth in association with the overgrowth component: hypoplasia of one limb has been described as a variant of classical phenotypes, accompanied by cutaneous signs [12]. Cases of undergrowth associated with lymphatic, venous or venous-capillary malformations, accompanied by variants in the *PIK3CA* gene, have been identified, highlighting this less-explored aspect alongside overgrowth in such conditions [13].

**Figure 1:**
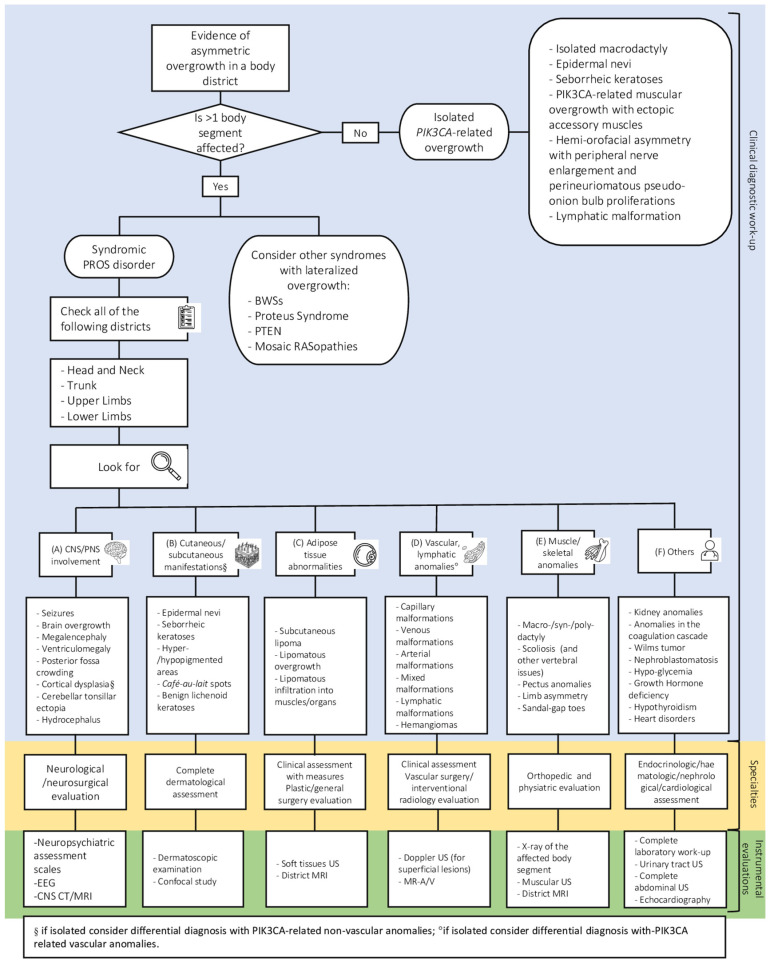
“Personalized follow-up strategy to be tailored according to the clinical and radiological features of patients with *PIK3CA*-related disorders. When the clinician is involved in the care of a patient showing an asymmetric overgrowth involving more than one body segment, they need to accurately check the following body district in order to speculate about a diagnosis of syndromic PROS: head and neck, trunk, four limbs [14]. The present flowchart provides general information about the different tissues and organs (see letters (A–E)) that can be affected and other possible issues (F) in a syndromic PROS. Moreover, in the boxes below, clinicians can find a detailed checklist of manifestations involving organs and tissues affected. According to the major clinical findings, different specialists may be included in the multidisciplinary evaluation and they may require further instrumental examination to support their diagnosis. PROS: *PIK3CA*-related Overgrowth syndrome; CNS: central nervous system; PNS: Peripheral nervous system. EEG: Electroencephalography; CT: computed tomography; MRI: magnetic resonance imaging; US: ultrasound; MR-A/V: magnetic resonance arteriography/venography. The Nominal Group Technique was used to generate the suggested strategy [5]”.

Less common manifestations are fetal macrosomia, growth retardation, feeding difficulties in infancy, facial asymmetry, dental crowding and other anomalies of dental eruption, epididymal cysts, and heart malformations. Laboratory abnormalities have also been reported. These include adrenal insufficiency, central hypothyroidism, growth hormone deficiency, hypoglycemia, coagulopathy (elevated D-dimer concentration, hypofibrinogenemia, and thrombocytopenia) (Figure 1).

**Figure 2:**
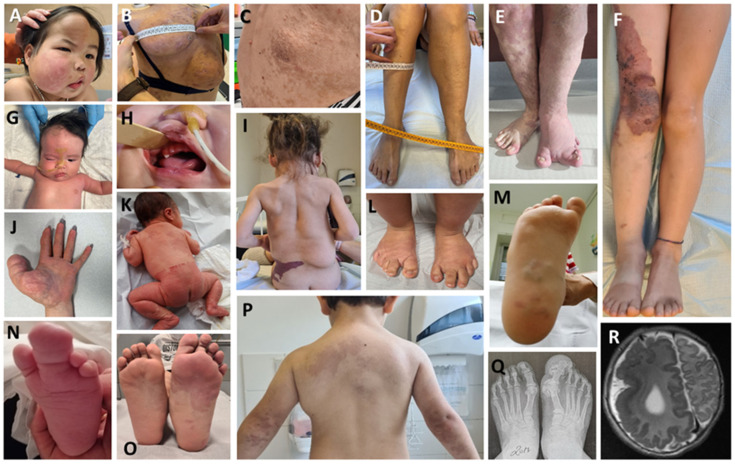
(**A**) Infiltrating fibro-lipomatous hyperplasia of right cheek and hemiface; (**B**–**D**) lipomatous malformation of the back (**B**) with seborrheic keratosis (**C**) and muscular hypertrophy of the left hemibody in the same patient (**C**); (**E**) Klippel–Trenaunay syndrome (KTS) with fibrose hyperplasia and vascular malformation of the left leg with splayed aspect and syndactyly of the second and third toes of the left foot; vascular malformation with macrodactyly of the fourth toe of the right foot; (**F**) Klippel–Trenaunay syndrome (KTS); (**G**,**H**) infiltrating fibro-lipomatosis of the face (**G**) with gingival hypertrophy, precocious tooth eruption and bilateral macrodontia (**H**); (**I**) congenital lipomatous overgrowth, vascular malformations, epidermal nevi and scoliosis/skeletal/spinal anomalies (CLOVES) with lipomatosis and vascular malformation of the trunk; (**J**) macrodactyly of the thumb and fibroadipose hyperplasia (FH) of the thumb and palm of the left hand; (**K**) diffuse capillary malformation with overgrowth (DCMO); (**L**) fibroadipose hyperplasia of the feet with splayed appearance in a patient with megalencephaly–capillary malformation (MCAP); (**M**) vascular malformation and FH of the sole of the right foot; (**N**) macrodactyly and syndactyly of the second and third toe and FH of the sole of the left foot; (**O**) fibrous hyperplasia with associated vascular malformation of the left foot; (**P**) CLOVES with lipomatosis and vascular malformation; (**Q**) X-ray of the feet showing bone and fibrous hyperplasia of the first toe of the left foot; (**R**) magnetic resonance imaging of the brain showing hemimegalencephaly and lissencephaly of the right hemisphere.

Despite recent revisions, the phenotypic classifications fail to cover all individuals due to the extreme variability of affected tissues (Figure 2). Therefore, the primary goal for clinicians dealing with *PIK3CA*-related disorders is to provide a patient-centered management and treatment plan, based on their specific needs.

## 3. Molecular Diagnosis

*PIK3CA* pathogenic variants are generally somatic, i.e., not present in all cells but detectable in DNA extracted from the affected tissue, so a biopsy of the affected site is often required for molecular diagnosis [7]. For this reason, molecular confirmation of the clinical diagnosis can be technically difficult in most cases. Molecular diagnosis requires (1) careful selection of the area of tissue to be biopsied (ideally a punch skin biopsy above the body area affected by overgrowth) and (2) high sensitivity of the analytical method to detecting variants even with a low degree of tissue mosaicism. The following guidelines may help to minimize the possibility of a false negative result.

### 3.1. Sampling

Analysis should preferably be performed on recently obtained and untreated sample rather than on cultured tissue. In clinical practice, it is commonly performed on tissue obtained during surgery or from an elective skin biopsy. The use of biopsy-derived fibroblast cultures should be avoided or limited to cases in which it is not possible to obtain a new biopsy sample since the presence of a pathogenic variant in *PIK3CA* may confer a selective advantage in vitro or vice versa; its absence could also be the result of a negative selection [7,15]. Therefore, the degree of mosaicism, if detected, or failure to identify the *PIK3CA* variant in cultured fibroblasts needs to be interpreted with caution. The use of pooled sampling (e.g., blood and a previous biopsy, or fibroblast from a scratch test) from the same subject may also be useful in detecting somatic genetic defects without the necessity for further confirmation. It is possible to screen for pathogenic variants in *PIK3CA* as well as in DNA extracted from blood or saliva, taking into account the rare cases of variants present at high levels of mosaicism or hypothetically germline. For example, in patients with MCAP, the use of this type of sample could represent the first step of a two-step strategy (biopsy only if blood and/or saliva are negative). 

### 3.2. Methods

It is preferable to use methods with high sensitivity in terms of limit of detection (LOD), which is the lowest variant allele frequency (VAF) that can be reliably detected. For NGS sequencing, LOD can vary depending on several factors, including the specific NGS platform, the depth of sequencing coverage, and the bioinformatics pipeline used for variant calling. Generally, NGS (next-generation sequencing) platforms utilizing targeted exomic multi-gene panels are capable of detecting variants with a limit of detection (LOD) as low as 5%, and this sensitivity can be further enhanced to even lower levels with a minimum read coverage of 1000X. Sanger sequencing is usually not able to detect variants with a VAF lower than 15–20% and has a low sensitivity compared to NGS methods [16]. This was recently highlighted in a review of more than 1000 PROS cases, as 10 hot-spot variants cover 70% of PROS cases [4]; therefore, a rapid screening of recurrent hotspot variants may be a reasonable initial diagnostic approach particularly in cases such as macrodactyly, CLOVES syndrome and KTS. However, since such restricted choice potentially leaves out (a) thirty percent of subjects (1 out of 3) potentially bearing different genetic defects, and (b) a number of identifiable gene defects directly involved in cellular and tissue growth control and involved in the PI3K upstream/downstream pathway, a targeted exome sequencing panel including the following genes is strongly suggested: *PIK3CA*, *CCND2*, *AKT1*, *AKT2*, *AKT3*, *PTEN*, *GNA11*, *GNAQ*, *RACE1*, *MTOR*, *PI3KCAa/d*, *PIK3R1*, *PIK3R2*, *PDK1*, *S6K1*, *TSC1*, *TSC2*, *UBF*, *TIF-IA*, *RPTOR*, *RICTOR*, *PI3KCD*, *TEK*, *CBL*, *RASA1*, *HRAS*, *KRAS*, *NRAS*, *SOS1*, *ARAF*, *CBL*, *RASA1*, *BRAF*, *MAP2K1*, *MEK*, and *PTPN11* [17,18].

### 3.3. Variants Validation

Variant identification should be followed, whenever possible, by validation using orthogonal methods such as Sanger sequencing for VAF ≥ 10%, pyrosequencing for VAF ≥ 5%, or ddPCR for VAF ≤ 5%. Repeating the NGS test with a read coverage of at least 1000X on the same sample could be a solution in all instances where an alternative method is unavailable or in cases of ambiguous variants with low VAF and suboptimal data quality. If multiple tissues of the same patient are tested, validation may not be necessary. If a pathogenic or probably pathogenic variant is identified, the description, interpretation of the variant and the bibliographic references should be included in the molecular report, whereas benign or probably benign variants should not be reported.

### 3.4. Negative Results and Further Considerations

A negative result is expected in a variable proportion of cases, ranging from 15 to 60%, as reported in recent papers on PROS cases [4]. This could be secondary to undetectable low level of mosaicism, the quality of the sample (especially in formalin-fixed paraffin-embedded tissue), the type and site of sampling, and the sensitivity of the analytical method used. Furthermore, a negative test does not exclude the clinical diagnosis, since it could be due to the presence of variants in genes that have not yet been identified. Finally, it is possible that a variant may be identified via whole exome or whole genome sequencing in patients in whom *PIK3CA*-related disorder has not been considered.

## 4. Follow-Up Strategies

### 4.1. Clinical Follow-Up

At first evaluation, patients with *PIK3CA*-related disorders should receive complete clinical and imaging assessment, with the aim of developing a proper personalized follow-up strategy according to their unique characteristics (Figure 1).

A personalized approach is mandatory, basing the follow-up protocol on the specific features of each individual than on the phenotypic sub-category they belong to [19]. At every medical evaluation, it is important that the patient undergo complete clinical evaluation, with particular attention to growth parameters. Besides height, weight, and cranial circumference (whose rapid increase may indicate some underlying brain anomaly), upper and lower extremities’ length and circumference should be measured, and if any limb length discrepancy is present, patients should be addressed to orthopedic evaluation to avoid functional consequences for gait, posture and the spinal column. In addition, scoliosis should always be clinically assessed. The clinician should also be careful when evaluating the abdomen, since masses and/or organomegaly may be detected. The trunk and the limbs should be checked thoroughly for the presence of lipomas; referral to dermatologists is important in case of skin lesions and vascular malformations (such as capillary malformations or venous ectasia); and mild hand and foot anomalies such as sandal gap or syndactyly can be also present. During childhood, routine neurodevelopmental assessment is suggested, focusing on psychomotor development and behavior. Furthermore, in case of onset of any kind of neurological disturbance (e.g., new onset seizures), the individual should be shortly referred to a neurologist [7]. Eye evaluation should follow regular monitoring as performed in the general population.

### 4.2. Radiological/Instrumental/Laboratory Follow-Up

The choice of imaging or laboratory tests should be determined on a case-by-case basis, taking into consideration factors such as the extent of the disease, its type, and its localization. In patients with neurological findings and/or overgrowth or CNS dysplasia, or in the presence of facial malformations, magnetic resonance imaging (MRI) of the brain and/or spine is recommended, as well as in case of rapid increase of cranial circumference; the procedure usually requires sedation in young children. The frequency of subsequent imaging will be scheduled case by case according to neurologists’ and neurosurgeons’ opinions. A magnetic resonance arteriography/venography (MR-A/V) of the chest, abdomen, pelvis, and limbs is suggested at diagnosis to check for deep vascular anomalies in specific districts affected by overgrowth. Doppler ultrasound (US) may be easily performed to check for and monitor the evolution of vascular anomalies in upper and lower limbs over time. In patients with extensive venous malformations (e.g., CLOVES syndrome, KTS), hematological or pediatric consultation is recommended to assess the risk of thrombosis and coagulopathy and for possible anticoagulant prophylaxis. In patients with overgrowth/undergrowth of a limited body area (e.g., a limb or a portion thereof), MRI at diagnosis is recommended to check what tissue is affected (muscle, bone, or others). As a general rule, more complex cases require periodic comprehensive reassessment every 6–12 months, depending on the rate of progression. A baseline screening for endocrinopathies is also suggested at first evaluation [7].

### 4.3. Tumor Surveillance

It is known that the PI3K-AKT-mTOR pathway has a key role in both physiological and malignant cell processes [20]. In detail, *PIK3CA* mutations are described in >10% of cancer patients and have been recognized as pathogenetic in many solid tumors such as breast, endometrial, bladder, colorectal, and head and neck squamous cell carcinoma, as well as in other benign skin lesions (e.g., epidermal nevi) [17,21]. It has recently been reported that pathogenic *PIK3CA* variants causing PROS are spread along the entire gene, but that the three hotspots most commonly mutated in cancer are involved in >50% of cases [4]. Given this proven relationship between *PIK3CA* mutations and cancer, a higher risk for malignancy would be expected in individuals with PROS. Nevertheless, in spite of this observation, cancer cases are neither consistently reported nor observed in clinical practice.

Most tumors identified in patients with *PIK3CA*-associated diseases are benign or locally invasive (e.g., kaposiform hemangioendothelioma [22]). Wilms’ tumors, or nephroblastoma, have been found with a frequency of 1.4–3.3%, in patients with CLOVES syndrome, MCAP, and KTS [23], as well as some anecdotal cases of leukemia, vestibular schwannoma [24], retinoblastoma, meningioma [25], and papillary intra-lymphatic angioendothelioma [26]. Data that clearly define an association between *PIK3CA*-related disorders and cancer development are limited; therefore, there is no international consensus on the appropriateness of oncological surveillance, particularly for Wilms’ tumors, in PROS patients. Some experts suggest following a schedule similar to that used for Beckwith–Wiedemann syndrome (every 3–4 months up to 8 years) [27], even though the risk is much lower. Others indicate surveillance every 5–6 months, believing that a timing of surveillance every 3 months is quite demanding for families in relation to the abovementioned limited oncological risk. It should however be taken into account that the time to tumor duplication in a Wilms’ tumor is approximately 30 days (ranging from 17 to 40 days) [28], and this longer interval may not translate into a real diagnostic gain. It seems reasonable to apply more stringent surveillance in situations where extensive involvement of mosaic tissue in the abdomen is suspected. It is advisable to define the surveillance strategy taking into account the needs and sensitivities of the family.

Anecdotally, tumors have not been observed in clinical practice. Based on these considerations, data are not sufficient to hypothesize a clear timing for surveillance protocol about this topic.

## 5. Therapeutic Approaches

### 5.1. Conventional Therapies

With the development of new diagnostic technologies, more and more overgrowth disorders are being classified as PROS, giving rise to a wide and clinically heterogeneous family of conditions. Due to the variable manifestations, multidisciplinary care is essential, possibly in a tertiary medical center where specialists have developed specific skills and experience in managing such complex conditions. Usually, a pediatric expert in the field of rare genetic conditions coordinates the management of these patients, in which many specialists are involved: geneticist, psychologist, orthopedic surgeon, dermatologist, neurologist, neurosurgeon, neuropsychiatrist, plastic surgeon, otolaryngologist, maxillofacial surgeon, vascular surgeon, physiatrist, physiotherapist, psychologist, speech therapist, pathologist, ophthalmologist, occupational therapist, neuro-psychomotor therapist, audiologist, dentist/orthodontist, prosthodontist, nutritionist, pulmonologist, and endocrinologist. Unfortunately, to date there is no resolutive treatment for these conditions, and there are still some aspects of care for which there is currently no shared indication from the scientific community [7]. As far as overgrowths/fibro-lipomatous masses are concerned, debulking surgery is usually the first choice in cases of functional limitations and/or pain. Many patients with *PIK3CA*-realted conditions in fact often complain about pain of variable intensity in multiple locations [29]. Huge demolitive procedures are often necessary, and depending on the involved area, sometimes more specialists need to be involved. The aim of the surgical treatment is to gain a good functional status in combination with the most aesthetically pleasing outcome possible. More than one procedure is often required, especially in the most severe cases with fast growing/recurrent masses or when there is vascular involvement [17]. An orthopedic surgeon should always be involved, given the high prevalence of skeletal anomalies in individuals with PROS, as lower limb length discrepancy, severe scoliosis, and progressive hand/foot macrodactyly may benefit from surgical treatment. Arthrodesis, debulking, epiphysiodesis, shortening osteotomy, resection of the phalanges, resection of the entire radius, and angular correction are some of the possible approaches with the aim of restoring or improving the patient’s functionality and appearance. Macrodactyly is a condition for which surgery can significantly enhance appearance; surgical interventions included debulking, ray resection, epiphysiodesis, and phalangeal resection. Multiple surgeries are often performed, especially for progressive overgrowth cases, with generally better outcomes observed for static macrodactyly [30]. However, individuals who undergo surgical treatment for macrodactyly might encounter a progression of tissue overgrowth later on in life, potentially leading to deformities such as joint ankyloses, new bone formation, and bony spurs [31]. Disagreement exists over the effectiveness of surgical methods for FIL treatment. Usually, partial resection is performed due to tumor infiltration in nearby tissues and its risky location for facial nerve function [32]. Early reports suggest prompt and wide excision, while others propose delayed resection to minimize nerve damage, procedures, and achieve balanced contours. Incomplete excision, especially in younger patients, poses the highest risk of re-growth [33,34]. Guided growth (i.e., a minimally invasive procedure for the selective temporary and reversible blockage of the growth plate of the affected bones) is the procedure of choice for cases with leg length discrepancy when shoe lift and/or insoles cannot be resolutive, as well as recommended for other genetic conditions such as Beckwith–Wiedemann syndrome [35]. Two studies involving patients with KTS examined the use of epiphysiodesis to treat LLD. Most patients showed improvement in LLD after surgery. Complications included issues such as loosening of epiphyseal staples, difficulties with wound healing, and instances of overcorrection leading to subsequent undergrowth in the treated leg. In general, the authors stated that operation surgery is warranted for LLD over 2.0 cm. Slight discrepancies (<2.0 cm) can be addressed by placing a lift in the opposite shoe if needed for symptoms. The timing of epiphysiodesis is crucial so that the affected and the unaffected extremities attain a similar length at skeletal maturity. Minor leg length discrepancies (less than 2.0 cm) can be managed via insertion of a lift in the contralateral shoe [30,36,37]. In the same way, it is of utmost importance that orthopedic surgeons and interventional radiologists refer patients with overgrowth to geneticists before performing surgery, in order to make a correct diagnosis and save tissue samples for molecular tests.

Neurosurgery might be considered in drug-resistant epilepsy, Chiari malformation and hydrocephalus.

In cases of vascular malformations, sclerotherapy, laser therapy, and oral medications are the treatments of choice. When lymphatic malformations are present, the main available treatment options are sclero-embolization and/or surgical excision. It is common agreement that the aim of the surgical procedure should be the morphological and functional maintenance of the affected body district [38]. Less invasive treatment options, such as sclerotherapy, are also available, with OK-432, doxycycline, bleomycin, bleomycin A5, ethanol, and sodio-tetradecyl-sulfate being some of the most used sclerotizing agents [39,40]. Its major complications are the deposits of scar tissue and swelling, with subsequent mass effect on closer structures. Other therapeutic possibilities are skin lasers of different wavelengths (depending on the type of lesion), endovascular lasers, manual lymphatic drainage, compression therapy, and cryotherapy. However, they can be accompanied by side effects and/or even important complications, therefore it is necessary to carry out a careful assessment, case by case, to stratify the risk/benefit ratio of any therapeutic strategy.

The presence of vascular malformations has been related to an increased risk of deep vein thrombosis and pulmonary embolism, especially in cases where there are combined capillary–lymphatic venous malformations, or in syndromes such as KTS and CLOVES. Additionally, anticoagulation is usually evaluated in high-risk situations or in case of prior DVT and not recommended routinely [41].

Finally, surgery is burdened by the recurrence of excessive growth in the operated areas and repeated surgery is common [7,42,43,44].

### 5.2. The Role of Rehabilitation Programs

A common complaint due to the involvement of the musculoskeletal system is the mobility impairment experienced by many patients, who may often require walking aids and struggle in autonomous ambulation for longer distances. That is why habilitative therapies such as physical therapy are fundamental to ensuring the well-being of patients during their adult years, to prevent deterioration of functional status and to improve quality of life. They should be started as early as possible with the aim of maintaining/improving muscle trophism and range of movement (especially when massive overgrowth affects the limbs). Orthopedics aids are generally useful together with habilitative therapies before and after surgical intervention to improve posture and gait pattern.

Exercises to maintain fine motor abilities, which may be jeopardized in case of macrodactyly, should also be included. Grip, handling, and writing impairment should be prevented or promptly treated.

### 5.3. Psychological Support

Given the extensive psychological burden that a PROS diagnosis—with all its consequences—implies, a psychologist/psychotherapist should most certainly be included in the multidisciplinary team taking care of the patient and also be involved in the evaluation of the family/support system. A complete psychological assessment should be performed on the affected individual and their family members/caregivers as soon as a diagnosis is confirmed with subsequent re-evaluation according to the situation.

Therapists can modulate their interventions according to the level of distress reported by the patient/their family.

Especially during the first few years of life and during childhood, interviews are directed towards the entire family system. During the transition to adulthood and especially in the teenage years, individual sessions should also be implemented to ensure the need for privacy and individualization is met according to the requirements of the involved individuals.

### 5.4. Pharmacological Therapies

In recent years, growing bodies of scientific data encompass the pathogenic role of the PI3K/AKT/mTOR pathway in human cancer. In detail, mutations in *PIK3CA* (especially affecting the p110α subunit) have been shown to be involved in the development of endometrial, breast, cervical, anal, head and neck, bladder, and colorectal cancer [45]. Consequently, small-molecule inhibitors of the pathway have been developed with the aim of treating the abovementioned and other malignancies. Such new drugs target the pathway at different levels, with inhibitors of PI3K, AKT, and mTOR [46].

Idelalisib, which targets the δ subunit of PI3K, was the first approved PI3K inhibitor developed for the treatment of relapsed hematological malignancies [47]. Subsequently, alpelisib (BYL719), a selective PI3Kα inhibitor, was approved for the treatment of advanced breast cancer [48]. Given the common genetic background, several molecules have been repurposed for individuals affected by PROS as new treatment opportunities [30]. A phase 1 (EPIK-P1) study of alpelisib for PROS patient has been completed (NCT04285723), while phase 2 and 3 trials are currently ongoing (NCT04589650 and NCT04980833 respectively). Venot et al. demonstrated the effectiveness of alpelisib in treating 17 patients with PROS, achieving a 100% response rate to treatment with 50 to 250 mg once daily with mild side effects (29.4% of cases, mostly hyperglycemia and gastrointestinal issues) and no serious adverse events [49]. Raghavendran et al. reported soft tissue and extremity bulk reduction, pain improvement, enhanced quality of life and functional abilities, with manageable side effects and favorable safety profile [50]. Alpelisib in children with *PIK3CA*-associated head and neck lymphatic malformations or FIL resulted in reduced lesion size, improved symptoms and function, led to avoidance of invasive procedures, and decreased facial volume [51]. A number of other reports on patients treated at various ages and with different phenotypes have been published thereon, with consistent results [43,52,53]. Alpelisib was also administered to patients with hemimegaloencephaly and West syndrome, showing partial beneficial effects on epilepsy [54].

Taselisib, a selective inhibitor of class I PI3K developed for breast cancer therapy [55], was investigated in the TOTEM study (NCT03290092) a phase 1/2 multicenter trial conducted on patients aged 16 to 65 years with PROS. In this study, although 76.4% of the participants reported clinical improvements, such as reduced pain, resolution of chronic bleeding, and functional enhancement, no significant reduction in affected tissue volume was observed. The reported side effects (mainly enteritis and pachymeningitis) were common, leading to the early termination of the trial for 2 out of the 19 patients enrolled. Therefore, despite the functional improvements, the safety profile of low-dose taselisib has prevented its long-term utilization [56].

Inhibitors targeting other key players have been investigated. For instance, the effect on PROS of miransertib administration, which targets AKT, has been reported in anecdotal cases. Notably, successful treatment was reported for a patient with Proteus syndrome and relapsed low-grade serous ovarian carcinoma, resulting in a significant improvement in their quality of life with miransertib [57]. It was administered to two patients with CLOVES and FIL with hemimegaloencephaly, with initial improvements and no significant toxicities; however, the treatment was discontinued due to a lack of sustained response and poor compliance [58]. Miransertib phase 1–2 clinical trial to assess the safety and tolerability of miransertib to participants aged at least 2 years with PROS or Proteus syndrome was terminated due to business reasons (NCT03094832).

Leoni et al. reported that low-dose oral treatment using the mTOR inhibitor sirolimus had positive effects on a child with fibroadipose hyperplasia (FAO) [59]. Sandbank et al. aimed to assess the effectiveness and safety of sirolimus for children and young adults with complicated vascular anomalies, finding that sirolimus led to clinical improvement in the majority of cases, making it a potentially successful and safe option for managing such cases. However, sirolimus led to several toxicities (including hypercholesterolemia, hypertriglyceridemia, elevated liver enzymes, mouth sores, thrombocytopenia, bacterial and opportunistic infections, and gastrointestinal issues), with a few patients experiencing interruptions in therapy due to adverse events [60]. A phase 2 clinical trial (NCT02428296) assessed the potential efficacy of low-dose sirolimus in patients with molecularly proven PROS, showing a moderate reduction in overgrown tissue volume after 26 weeks: this study, however, emphasizes that the side-effect profile is noteworthy, as nearly three-quarters of participants encountered at least one adverse event linked to sirolimus, of which roughly one-third reached grade 3 or 4 in severity requiring hospitalization and including infections, neutropenia, interstitial pneumonitis, and sirolimus hypersensitivity syndrome. This resulted in a substantial number of patients choosing to withdraw from the study [44]. Also, Wiegand et al. observed that sirolimus ameliorated symptoms and decreased the size of lymphatic malformations in children with extensive head and neck involvement (91% of exhibited positive responses), but noted that such side effects were frequently observed, suggesting that the decision to administer sirolimus should be multidisciplinary and weighed on each case [38].

Unfortunately, to date no pharmacological treatment has been officially approved in Europe, whereas in the US, after EPIK-P1 trial (NCT04285723), only alpelisib has been approved by the FDA for individuals ≥ 2 years old with severe PROS [49,50]. Moreover, in other countries, managed access programs are available to provide expanded access to individuals or groups of patients with disabling symptoms due to PROS who do not have any other chance of treatment. Although these drugs represent an interesting opportunity, there are still many concerns and unanswered questions about their use. Whilst therapies have shown some promise in regards to tissue regression and cessation of growth, these drugs are not always well tolerated. Therefore, further studies will be necessary to determine which patients may benefit most from such therapies according to a careful individualized risk–benefit assessment, to define optimal dosing and the appropriate timing of treatment.

## 6. Functional Assessment

As for many other multisystem disorders, patients with PROS conditions require highly complex care, especially in the cases with the most severe phenotypes. An integrated approach is often required, including home paramedical support and close collaboration between the reference center and the local medical services.

It is of utmost importance to maintain and, whenever possible, to improve the physical, mental, and social status of the patient, with the aim to reach the best possible quality of life for the individuals and their support systems. A comprehensive functional assessment should always be obtained, to create an integrated system involving school, habilitative centers, and other local medical/assistance services. The early initiation of appropriate, meaningful, and effective teaching and learning activities, the development of an individualized education plan in collaboration with special needs teachers and pediatric neurologists, are important support tools for patients’ development. Psychologists/psychotherapists also represent an important aid for families to guide habilitative and therapeutic decisions [61].

## 7. Transition to Adulthood

Transition to adulthood is a great challenge for most individuals affected by rare genetic conditions, as well as for people with PROS. The lack of a specialized care manager figure (such as a pediatrician with expertise in the field of rare disorders is during childhood) is a common complaint among patients reaching the age of majority. In fact, primary care physicians do not usually have the capacity to manage such complex conditions only in the setting of local health services. This shortfall may be partly explained by the fact that rare and ultra-rare disorders are a relative novelty for adult medicine due to the improvement of care and treatment protocols for these conditions and to the increase of children surviving into adulthood.

Currently, pediatric centers provide, as far as possible, the necessary support during the transition to adulthood, often taking care of individuals far beyond pediatric age.

However, the adult patient has specific clinical characteristics and care needs that are very different from those of children. It is therefore necessary to invest in the training of adult physicians and primary care physicians so that, in the coming years, they can develop specific skills in this area and become care managers of the multidisciplinary assessment for adults with rare disorders and complex care needs.

## 8. Italian Law—Conclusions

As set out in the provisions of the Decree of the President of the Council of Ministers of 12 January 2017, in Italy, individuals with *PIK3CA*-related conditions (as well as people living with other rare/disabling disorders) receive free medical assistance via the NHS for any procedure (diagnostic/treatment/follow-up protocols) related to their rare disease.

They can also be granted free access to pharmacological therapies before their marketing authorization from the Italian Medicines Agency (AIFA) or to off-label treatments via various early-access tools besides the expanded access/compassionate use programs. Moreover, especially in the most disabling cases, patients have specific law-warranted rights, having access to various benefits and allowances. Parallelly, similar facilitations have been instituted in many countries.

Despite the available resources, patients with PROS often encounter many challenges in everyday life besides disease-related issues. The risk of receiving a delayed diagnosis and the difficult task of finding the right care team are only some of the first issues to arise, followed by the difficult management of symptoms and the lack of effective therapeutic options [61]. Furthermore, sporadic case reports of patients with germline variants in *PIK3CA* are not sufficient to obtain enough evidence to justify surveillance protocols [62,63].

It is indeed essential that these individuals be referred to expert physicians as soon as possible, so that personalized diagnostic and follow-up strategies according to the individual’s needs can be promptly developed. In this context, a network of specialists is essential to expedite the diagnosis and to provide the most complete multidisciplinary care aimed at avoiding delayed/incorrect diagnosis, inappropriate treatments, and unnecessary mobility for patients looking for second opinions and alternative treatment possibilities. Furthermore, given the large phenotypic variability of *PIK3CA*-related conditions, an individualized approach should be offered to any patient. Moreover, patients and their family members need to be sufficiently empowered, and communication from their care team should be as effective and empathetic as possible, so that they can be actively involved in the decision-making process and their preferences can be valued, aimed at developing a patient-clinician partnership and trusting relationship, thus improving adherence to medical and lifestyle recommendations, and maximizing the health outcomes.

Nevertheless, managing these patients remains challenging because patients’ needs and health issues are enormously fragmented and the natural history of this group of conditions is still largely unknown. International networking and collaboration between various stakeholders, including advocacy groups, is therefore key in recognizing unmet needs and research priorities to improve the quality of life and care of those living with *PIK3CA*-related conditions.

## References

[1] Reynolds G., Cardaropoli S., Carli D., Luca M., Gazzin A., Coppo P., La Selva R., Piglionica M., Bagnulo R., Turchiano A. (2023). Epidemiology of the disorders of the Pik3ca-related overgrowth spectrum (Pros). Eur. J. Hum. Genet..

[2] Canaud G., Hammill A.M., Adams D., Vikkula M., Keppler-Noreuil K.M. (2021). A review of mechanisms of disease across PIK3CA-related disorders with vascular manifestations. Orphanet J. Rare Dis..

[3] Mirzaa G., Timms A.E., Conti V., Boyle E.A., Girisha K.M., Martin B., Kircher M., Olds C., Juusola J., Collins S. (2016). PIK3CA-associated developmental disorders exhibit distinct classes of mutations with variable expression and tissue distribution. JCI Insight.

[4] Mussa A., Leoni C., Iacoviello M., Carli D., Ranieri C., Pantaleo A., Buonuomo P.S., Bagnulo R., Ferrero G.B., Bartuli A. (2022). Genotypes and phenotypes heterogeneity in PIK3CA-related overgrowth spectrum and overlapping conditions: 150 novel patients and systematic review of 1007 patients with PIK3CA pathogenetic variants. J. Med. Genet..

[5] Søndergaard E., Ertmann R.K., Reventlow S., Lykke K. (2018). Using a modified nominal group technique to develop general practice. BMC Fam. Pract..

[6] Keppler-Noreuil K.M., Rios J.J., Parker V.E.R., Semple R.K., Lindhurst M.J., Sapp J.C., Alomari A., Ezaki M., Dobyns W., Biesecker L.G. (2015). PIK3CA-related overgrowth spectrum (PROS): Diagnostic and testing eligibility criteria, differential diagnosis, and evaluation. Am. J. Med. Genet. Part A.

[7] Douzgou S., Rawson M., Baselga E., Danielpour M., Faivre L., Kashanian A., Keppler-Noreuil K.M., Kuentz P., Mancini G.M.S., Maniere M.C. (2022). A standard of care for individuals with PIK3CA-related disorders: An international expert consensus statement. Clin. Genet..

[8] Carli D., De Pellegrin M., Franceschi L., Zinali F., Paonessa M., Spolaore S., Cardaropoli S., Cravino M., Marcucci L., Andreacchio A. (2021). Evolution over Time of Leg Length Discrepancy in Patients with Syndromic and Isolated Lateralized Overgrowth. J. Pediatr..

[9] Durmaz E.Ö., Demircioğlu D., Dikmen P.Y., Alanay Y., Alanay A., Demirkesen C., Tokat F., Karaarslan E. (2022). A Review on Cutaneous and Musculoskeletal Manifestations of CLOVES Syndrome. Clin. Cosmet. Investig. Dermatol..

[10] Stillo F., Diociaiuti A., Neri I., Mattassi R., Baraldini V., Dalmonte P., Amato B., Ametrano O., Amico G., Bianchini G. (2022). Guidelines for Vascular Anomalies General Coordinators. Int. Angiol..

[11] Kunimoto K., Yamamoto Y., Jinnin M. (2022). ISSVA Classification of Vascular Anomalies and Molecular Biology. Int. J. Mol. Sci..

[12] Triana P., Sarmiento M.d.C., Rodriguez-Laguna L., Martinez-Glez V., Lopez-Gutierrez J.C. (2023). Undergrowth of First Toe in PiK3CA-Related Overgrowth Spectrum (PROS). Ann. Vasc. Surg..

[13] Martinez-Glez V., Rodriguez-Laguna L., Viana-Huete V., García Torrijos C., Hurtado B., Lapunzina P., Triana P., López-Gutiérrez J.C. (2022). Segmental undergrowth is associated with pathogenic variants in vascular malformation genes: A retrospective case-series study. Clin. Genet..

[14] Mussa A., Carli D., Cardaropoli S., Ferrero G.B., Resta N. (2021). Lateralized and segmental overgrowth in children. Cancers.

[15] Kuentz P., St-Onge J., Duffourd Y., Courcet J.B., Carmignac V., Jouan T., Sorlin A., Abasq-Thomas C., Albuisson J., Amiel J. (2017). Molecular diagnosis of PIK3CA-related overgrowth spectrum (PROS) in 162 patients and recommendations for genetic testing. Genet. Med..

[16] Singh R.R. (2020). Next-Generation Sequencing in High-Sensitive Detection of Mutations in Tumors: Challenges, Advances, and Applications. J. Mol. Diagn..

[17] Madsen R.R., Vanhaesebroeck B., Semple R.K. (2018). Cancer-Associated PIK3CA Mutations in Overgrowth Disorders. Trends Mol. Med..

[18] Scalia P., Williams S.J., Fujita-Yamaguchi Y., Giordano A. (2023). Cell cycle control by the insulin-like growth factor signal: At the crossroad between cell growth and mitotic regulation. Cell Cycle.

[19] Rodríguez-Laguna L., Davis K., Finger M., Aubel D., Vlamis R., Johnson C. (2022). Mapping the PIK3CA-related overgrowth spectrum (PROS) patient and caregiver journey using a patient-centered approach. Orphanet J. Rare Dis..

[20] Samuels Y., Wang Z., Bardelli A., Silliman N., Ptak J., Szabo S., Yan H., Gazdar A., Powell S.M., Riggins G.J. (2004). High Frequency of Mutations of the PIK3CA Gene in Human Cancers. Science.

[21] Tharin Z., Richard C., Derangère V., Ilie A., Arnould L., Ghiringhelli F., Boidot R., Ladoire S. (2023). PIK3CA and PIK3R1 tumor mutational landscape in a pan-cancer patient cohort and its association with pathway activation and treatment efficacy. Sci. Rep..

[22] Carli D., Kalantari S., Manicone R., Coppo P., Francia di Celle P., La Selva R., Santoro F., Ranieri C., Cardaropoli S., Fagioli F. (2021). Kaposiform hemangioendothelioma further broadens the phenotype of PIK3CA-related overgrowth spectrum. Clin. Genet..

[23] Gripp K.W., Baker L., Kandula V., Conard K., Scavina M., Napoli J.A., Griffin G.C., Thacker M., Knox R.G., Clark G.R. (2016). Nephroblastomatosis or Wilms tumor in a fourth patient with a somatic PIK3CA mutation. Am. J. Med. Genet. Part A.

[24] Mills J.R., Moyer A.M., Kipp B.R., Poplawski A.B., Messiaen L.M., Babovic-Vuksanovic D. (2018). Unilateral vestibular schwannoma and meningiomas in a patient with PIK3CA-related segmental overgrowth: Co-occurrence of mosaicism for 2 rare disorders. Clin. Genet..

[25] Griff J.R., Duffy K.A., Kalish J.M. (2020). Characterization and Childhood Tumor Risk Assessment of Genetic and Epigenetic Syndromes Associated with Lateralized Overgrowth. Front. Pediatr..

[26] Debelenko L., Mansukhani M.M., Remotti F. (2023). Papillary Intralymphatic Angioendothelioma in a Child with PIK3CA-Related Overgrowth Spectrum: Implication of PI3K Pathway in the Vascular Tumorigenesis. Pediatr. Dev. Pathol..

[27] Brioude F., Kalish J.M., Mussa A., Foster A.C., Bliek J., Ferrero G.B., Boonen S.E., Cole T., Baker R., Bertoletti M. (2018). Clinical and molecular diagnosis, screening and management of Beckwith–Wiedemann syndrome: An international consensus statement. Nat. Rev. Endocrinol..

[28] Craft A.W. (1999). Growth rate of Wilms’ tumour. Lancet.

[29] Raymond K., Vallow S., Saucier C., Jackson K., White M.K., Lovley A., D’Alessio D. (2022). Qualitative research with patients and caregivers of patients with PIK3CA related overgrowth spectrum: Content validity of clinical outcome assessments. J. Patient-Rep. Outcomes.

[30] Bernhard S.M., Adam L., Atef H., Häberli D., Bramer W.M., Minder B., Döring Y., Laine J.E., Muka T., Rössler J. (2022). A systematic review of the safety and efficacy of currently used treatment modalities in the treatment of patients with PIK3CA-related overgrowth spectrum. J. Vasc. Surg. Venous Lymphat. Disord..

[31] Stor M.L.E., Lokhorst M.M., Horbach S.E.R., van der Horst C.M.A.M. (2022). The long-term progression of macrodactyly. JPRAS Open.

[32] Singh K., Sen P., Musgrove B.T., Thakker N. (2011). Facial infiltrating lipomatosis: A case report and review of literature. Int. J. Surg. Case Rep..

[33] Kamal D., Breton P., Bouletreau P. (2010). Congenital infiltrating lipomatosis of the face: Report of three cases and review of the literature. J. Cranio-Maxillofac. Surg..

[34] Kang N., Ross D., Harrison D. (1998). Unilateral hypertrophy of the face associated with infiltrating lipomatosis. J. Oral Maxillofac. Surg..

[35] De Pellegrin M., Brogioni L., Laskow G., Barera G., Pajno R., Osimani S., Russo S., Marcucci L. (2021). Guided growth in leg length discrepancy in beckwith-wiedemann syndrome: A consecutive case series. Children.

[36] Jacob A.G., Driscoll D.J., Shaughnessy W.J., Stanson A.W., Clay R.P., Gloviczki P. (1998). Klippel-Trénaunay syndrome: Spectrum and management. Mayo Clin. Proc..

[37] Raab P., Wild A., Seller K., Krauspe R. (2001). Correction of length discrepancies and angular deformities of the leg by Blount’s epiphyseal stapling. Eur. J. Pediatr..

[38] Wiegand S., Wichmann G., DIetz A. (2018). Treatment of Lymphatic Malformations with the mTOR Inhibitor Sirolimus: A Systematic Review. Lymphat. Res. Biol..

[39] Lee S.Y., Loll E.G., Hassan A.E.S., Cheng M., Wang A., Farmer D.L. (2022). Genetic and Molecular Determinants of Lymphatic Malformations: Potential Targets for Therapy. J. Dev. Biol..

[40] Snyder E.J., Sarma A., Borst A.J., Tekes A. (2022). Lymphatic Anomalies in Children: Update on Imaging Diagnosis, Genetics, and Treatment. AJR. Am. J. Roentgenol..

[41] Keppler-Noreuil K.M., Lozier J., Oden N., Taneja A., Burton-Akright J., Sapp J.C., Biesecker L.G. (2019). Thrombosis risk factors in PIK3CA-related overgrowth spectrum and Proteus syndrome. Am. J. Med. Genet. Part C Semin. Med. Genet..

[42] Maconi G., Parente F., Bollani S., Cesana B., Porro G., Bianchi E. (1996). Abdominal Ultrasound in the Assessment of Extent and Activity of Crohn’s Disease: Clinical Significance and Implication of Bowel Wall Thickening. Am. J. Gastroenterol..

[43] Pagliazzi A., Oranges T., Traficante G., Trapani C., Facchini F., Martin A., Semeraro A., Perrone A., Filippeschi C., Giglio S. (2021). PIK3CA-Related Overgrowth Spectrum from Diagnosis to Targeted Therapy: A Case of CLOVES Syndrome Treated with Alpelisib. Front. Pediatr..

[44] Parker V.E.R., Keppler-Noreuil K.M., Faivre L., Luu M., Oden N.L., De Silva L., Sapp J.C., Andrews K., Bardou M., Chen K.Y. (2019). Safety and efficacy of low-dose sirolimus in the PIK3CA-related overgrowth spectrum. Genet. Med..

[45] Zehir A., Benayed R., Shah R.H., Syed A., Middha S., Kim H.R., Srinivasan P., Gao J., Chakravarty D., Devlin S.M. (2017). Mutational landscape of metastatic cancer revealed from prospective clinical sequencing of 10,000 patients. Nat. Med..

[46] Belli C., Repetto M., Anand S., Porta C., Subbiah V., Curigliano G. (2023). The emerging role of PI3K inhibitors for solid tumour treatment and beyond. Br. J. Cancer.

[47] Brown J.R., Byrd J.C., Coutre S.E., Benson D.M., Flinn I.W., Wagner-Johnston N.D., Spurgeon S.E., Kahl B.S., Bello C., Webb H.K. (2014). Idelalisib, an inhibitor of phosphatidylinositol 3-kinase p110δ, for relapsed/refractory chronic lymphocytic leukemia. Blood.

[48] André F., Ciruelos E., Rubovszky G., Campone M., Loibl S., Rugo H.S., Iwata H., Conte P., Mayer I.A., Kaufman B. (2019). Alpelisib for PIK3CA -Mutated, Hormone Receptor–Positive Advanced Breast Cancer. N. Engl. J. Med..

[49] Venot Q., Blanc T., Rabia S.H., Berteloot L., Ladraa S., Duong J.P., Blanc E., Johnson S.C., Hoguin C., Boccara O. (2018). Targeted therapy in patients with PIK3CA-related overgrowth syndrome. Nature.

[50] Raghavendran P., Albers S.E., Phillips J.D., Zarnegar-Lumley S., Borst A.J. (2022). Clinical Response to PI3K-α Inhibition in a Cohort of Children and Adults with PIK3CA-Related Overgrowth Spectrum Disorders. J. Vasc. Anomalies.

[51] Wenger T., Perkins J., Drusin M., Bindschadler M., Bly R., Friedman S., Bonilla-Velez J., Dahl J., Ganti S., Mercan E. (2022). eP282: Alpelisib for the treatment of PIK3CA-related head and neck lymphatic malformations and overgrowth. Genet. Med..

[52] Garneau A.P., Haydock L., Tremblay L.E., Isenring P. (2021). Somatic non-cancerous PIK3CA-related overgrowth syndrome treated with alpelisib in North America. J. Mol. Med..

[53] López Gutiérrez J.C., Lizarraga R., Delgado C., Martínez Urrutia M.J., Díaz M., Miguel M., Triana P. (2019). Alpelisib Treatment for Genital Vascular Malformation in a Patient with Congenital Lipomatous Overgrowth, Vascular Malformations, Epidermal Nevi, and Spinal/Skeletal Anomalies and/or Scoliosis (CLOVES) Syndrome. J. Pediatr. Adolesc. Gynecol..

[54] Morin G., Degrugillier-Chopinet C., Vincent M., Fraissenon A., Aubert H., Chapelle C., Hoguin C., Dubos F., Catteau B., Petit F. (2022). Treatment of two infants with PIK3CA-related overgrowth spectrum by alpelisib. J. Exp. Med..

[55] Dickler M.N., Saura C., Richards D.A., Krop I.E., Cervantes A., Bedard P.L., Patel M.R., Pusztai L., Oliveira M., Cardenas A.K. (2018). Phase II Study of Taselisib (GDC-0032) in Combination with Fulvestrant in Patients with HER2-Negative, Hormone Receptor–Positive Advanced Breast Cancer. Clin. Cancer Res..

[56] Luu M., Vabres P., Devilliers H., Loffroy R., Phan A., Martin L., Morice-Picard F., Petit F., Willems M., Bessis D. (2021). Safety and efficacy of low-dose PI3K inhibitor taselisib in adult patients with CLOVES and Klippel–Trenaunay syndrome (KTS): The TOTEM trial, a phase 1/2 multicenter, open-label, single-arm study. Genet. Med..

[57] Leoni C., Gullo G., Resta N., Fagotti A., Onesimo R., Schwartz B., Kazakin J., Abbadessa G., Crown J., Collins C.D. (2019). First evidence of a therapeutic effect of miransertib in a teenager with Proteus syndrome and ovarian carcinoma. Am. J. Med. Genet. Part A.

[58] Forde K., Resta N., Ranieri C., Rea D., Kubassova O., Hinton M., Andrews K.A., Semple R., Irvine A.D., Dvorakova V. (2021). Clinical experience with the AKT1 inhibitor miransertib in two children with PIK3CA-related overgrowth syndrome. Orphanet J. Rare Dis..

[59] Leoni C., Onesimo R., Resta N., Patti M.L., De Santis R., Bagnulo R., Russo L., Manfredi R., Genuardi M., Zampino G. (2019). Old treatments for new genetic conditions: Sirolimus therapy in a child affected by mosaic overgrowth with fibroadipose hyperplasia. Clin. Genet..

[60] Sandbank S., Molho-Pessach V., Farkas A., Barzilai A., Greenberger S. (2019). Oral and topical sirolimus for vascular anomalies: A multicentre study and review. Acta Derm. Venereol..

[61] Dexheimer J., Mirzaa G.M. (2022). Brain Abnormalities in PIK3CA-Related Overgrowth Spectrum: Physician, Patient, and Caregiver Experiences. Adv. Ther..

[62] Bourgon N., Carmignac V., Sorlin A., Duffourd Y., Philippe C., Thauvin-Robinet C., Guibaud L., Faivre L., Vabres P., Kuentz P. (2022). Clinical and molecular data in cases of prenatal localized overgrowth disorder: Major implication of genetic variants in PI3K-AKT-mTOR signaling pathway. Ultrasound Obstet. Gynecol..

[63] Zollino M., Ranieri C., Grossi V., Leoni C., Lattante S., Mazzà D., Simone C., Resta N. (2019). Germline pathogenic variant in PIK3CA leading to symmetrical overgrowth with marked macrocephaly and mild global developmental delay. Mol. Genet. Genom. Med..

